# Psychological responses of Tunisian general population during COVID-19 pandemic

**DOI:** 10.11604/pamj.2021.40.74.26379

**Published:** 2021-10-04

**Authors:** Wiem Bouattour, Mariem Turki, Sahar Ellouze, Neila Messedi, Fadwa Charfeddine, Najla Halouani, Lobna Aribi, Jihen Aloulou

**Affiliations:** 1 Psychiatry B Department, Hedi Chaker University Hospital, Sfax, Tunisia

**Keywords:** COVID-19, pandemic, anxiety, depression, insomnia, mental health

## Abstract

**Introduction:**

this is the first study assessing the psychological impact on Tunisian general population during the first peak of the COVID-19 pandemic. We aimed to assess the prevalence of anxiety, depressive symptoms and insomnia, as well as associated factors.

**Methods:**

a cross-sectional study was conducted through an online survey of 1615 people during March 23^rd^ to May 5^th^, 2020. We used the hospital anxiety and depression scale for anxiety and depression screening, and the insomnia severity index to assess sleep disturbances.

**Results:**

our study revealed a high prevalence of anxiety and depressive symptoms, and insomnia (70.9%, 71.1% and 60.6% respectively). Multivariable statistics showed that anxiety symptoms were correlated to female gender (adjusted odds ratio [aOR] 1.784, 95% confidence interval (CI 1.252-2.542; p=10^-3^), history of mental illness (aOR: 1.680, 95% CI: 1.329-2.125; p<10^-3^), frequently social media exposure (aOR: 1.578, 95%CI: 1.250-1.992; p<10^-3^), times to focus on COVID-19 ≥ 3hours (aOR: 1.840, 95% CI: 1.433-2.362; p<10^-3^), consultation with doctor in the clinic in the past 14 days (aOR: 1.821, 95%CI : 1.220-2.718; p=0.003) and recent traumatic event in the past 14 days (aOR: 1.641,95% CI: 1.331-2.024; p<10^-3^). Principal factors associated with depressive symptoms included female gender (aOR: 1.637, 95% CI: 1.150-2.331; p=0.006), history of mental illness (aOR: 1.498, 95% CI: 1.189-1.888; p=10^-3^), times to focus on COVID-19 ≥ 3hours (aOR: 1.956, 95% CI: 1.555-2.461; p<10^-3^), and recent traumatic event in the past 14 days (aOR: 1.558, 95% CI: 1.265-1.919; p<10^-3^). The main factors correlated to insomnia were younger (age <35years) (aOR: 1.592, 95% CI: 1.17 -2.152; p=0.003), female gender (aOR: 1.864, 95% CI: 1.252-2.775; p=0.002), having organic diseases (aOR: 1.527, 95% CI: 1.131-2.061; p=0.006), history of mental illness (aOR: 1.777, 95% CI: 1.396-2.263; p<10^-3^), students (aOR: 1.931, 95% CI: 1.495-2.495; p<10^-3^), times to focus on COVID-19 ≥3hours (aOR: 1.877, 95% CI: 1.467-2.400; p<10^-3^) and recent traumatic event (aOR: 1.431, 95% CI: 1.144-1.789; p=0.002).

**Conclusion:**

our study revealed a major mental health burden in Tunisia during COVID-19 pandemic. Many factors were correlated to anxiety, depressive symptoms and insomnia, suggesting the need for greater psychological support in general and in certain vulnerable groups.

## Introduction

The novel severe acute respiratory syndrome coronavirus 2 (SARS-CoV-2), previously provisionally named Coronavirus disease 2019 (COVID-19), was first identified in China (city of Wuhan) at the end of 2019 [1]. In less than 3 months, COVID-19 has spread rapidly around the world, affecting 210 countries and causing more than 100,000 deaths [2]. On 30^th^ January 2020, World Health Organization (WHO) described SARS-CoV-2 as Public Health Emergency of International concern [2] and on March 11, it was declared as a pandemic [3]. The COVID-19 epidemic has been officially developing in Tunisia since March 2^nd^, 2020 [4]. In view of global data and in particular taking into account the effect of COVID-19 on Mediterranean countries, the Tunisian government has taken several anticipatory measures to prevent the progression of the pandemic [4]. Tunisia started containment almost 2 weeks earlier given the level of pandemic spread. This approach aims to reverse epidemic growth, by reducing the number of cases to low levels through social distancing [5]. According to official sources during the period of the study (March 23^rd^- May 5^th^, 2020), the number of positive results for COVID-19 rose from 114 to 1025, and the number of deaths rose from 3 to 43 [6]. People's responses to epidemics are diverse [7]. The study of risk factors and moderators can help to better understand the development and maintenance of psychological symptoms and to develop preventive measures and targeted therapeutic interventions. This is the first study in the scientific literature to report the psychological impact of COVID-19 on a sample of the Tunisian population starting three weeks after the outbreak of the epidemic. This study aimed to determine the prevalence of psychological problems, including anxiety, depressive symptoms and insomnia, and associated factors among Tunisians during the peak of the COVID-19 epidemic with strict containment measures applied to most of the population.

## Methods

**Study design and setting:** we conducted a cross-sectional survey to assess the Tunisian's psychological response during the epidemic of COVID-19. Data were collected between March 23^rd^ and May 5^th^, 2020 using an online questionnaire spread throughout social media (Facebook).

**Study population:** our questionnaire was distributed via social networks (Facebook) and was intended for all adult Tunisian citizens (general population). Responses of participants under the age of 18 years, foreigners, and questionnaires not filled-in completely were excluded. In sum, the study received a total of 1670 valid responses, from which 55 were excluded (foreigner participants). Consequently, only 1615 responses were included.

**Data collection and definitions:** the online questionnaire was preceded by a brief introduction informing the participants about the objectives of the study, its confidentiality and the use of data only for research purposes. The structured questionnaire consisted of questions covering several areas; demographic data (age, gender, marital status, educational level, living areas, employment status), history of organic/mental disease, substance use, clinical profile in the past 14 days, recent quarantine, recent traumatic event in the past 14 days. Satisfaction with information received regarding the pandemic, social media exposure and mental health status. Social media exposure was measured by asking respondents: i) how often during the past week they were exposed to news and information about COVID-19 on social media. Response options were “less”, “sometimes” and “frequently” ii) How many hours per day (<1, 1-2, ≥ 3) they spent receiving information about COVID-19 from different media sources in the past one week [8]. Mental health status was measured using the Arabic version of the Hospital anxiety and depression scale (HADS) [9] and the French version of the 7-item insomnia severity index (ISI) [10]. The HADS, developed 30 years ago by Zigmond and Snaith [11], is a 14-item scale, which has been validated in many languages, countries and settings including community settings [12]. It consists of two subscales, each containing seven items on a 4-point Likert scale (ranging from 0 to 3). Participants were told that the questions asked were related to their mental state during the last 2 weeks. Hospital anxiety and depression scale is scored by summing up the ratings for the 14 items to yield a total score, and by summing up the ratings for the 7 items of each subscale to yield separate scores for anxiety and depression. Two cut-off scores are validated to identify anxiety and depression: 11 for anxiety/depression case and 8 for anxiety/depression borderline case. The ISI is a seven-question instrument assessing a current (i.e. preceding 2 weeks) sleep problem. It has been shown to be valid and reliable [13]. The first three items evaluate the severity of sleep onset, sleep maintenance, and early morning awakening problems. Subsequent items assess the degree of satisfaction or dissatisfaction with the current sleep problem, interference with daily functioning, noticeability of impairment attributed to the sleep problem, and amount of worry due to the current sleep problem. Items are rated on a five-point Likert scale (‘0´ representing none or not at all and '4´ representing very much). Total scores range from 0 to 28 and are categorized as follows: normal (0-7), sub- threshold (8-14), moderate insomnia (15-21), or severe insomnia (22-28) [14].

**Statistical analyses:** statistical analysis was performed using Statistical package for the social sciences (SPSS) 22.0 (IBM SPSS Statistics, New York, United States). The results of quantitative variables were presented as mean ± Standard deviation (SD). Those of qualitative variables were presented as numbers and percentages. Then, all variables that had p≤0.2 in the univariate analysis were entered into a multivariate model using backward linear regression analysis to identify independent factors of anxiety and depressive symptoms, as well as insomnia. Data were considered statistically significant when p < 0.05.

**Ethical considerations:** electronic informed consent was requested from each participant before starting the survey. The study was conducted in accordance with the Declaration of Helsinki, and the protocol was approved by the “Committee for the Protection of Persons” of the University of Sfax, Tunisia (Project identification code “CPP SUD N° 0256/2020”).

## Results

**Demographic characteristics:** in total, 1615 Tunisian adults completed the questionnaire; their basic characteristics are shown in Table 1. The average age was 30.5 years (±9.41). Most participants were women (88.7%), aged less than 35 years (71.5%), unmarried (63%), and had high school level (94.1%). Among participants, 51.8% had a regular work, 7.4% were health workers, 27.2% were smoking, 16.5% consumed alcohol, 17.3% had organic disease and 28.2% had history of mental illness.

**Table 1:** baseline characteristics of respondents (n = 1615)

Characteristic		Mean (SD) or n (%)
**Age**	< 35years	30.5 (±9.41) 1155 (71.5)
	≥ 35 years	460 (28.5)
**Gender**	Male	183 (11.5)
	Female	1432 (88.7)
**Marital status**	Married	598 (37)
	Unmarried	1017 (63)
**Educational level**	Second school level	95 (5.9)
	High school level	1520 (94.1)
**Living areas**	North	1101 (68.2)
	Center	260 (16.1)
	South	254 (15.7)
**Having organic diseases**	Yes	279 (17.3)
	No	1336 (82.7)
	Diabetes	40 (2.5)
	High blood pressure	43 (2.7)
	Heart disease	18 (1.1)
	Chronic respiratory pathology	103 (6.4)
	Others	90 (5.5)
**History of mental illness**	Yes	455 (28.2)
	No	1160 (71.8)
	Anxiety disorder	308 (19.1)
	Depressive disorder	210 (13)
	Bipolar disorder	44 (2.7)
	Suicide attempt	41 (2.5)
	Others	7 (0.4)
**Smoking**	Yes	440 (27.2)
	No	1175 (72.8)
**Alcohol consumption**	Yes	267 (16.5)
	No	1348 (83.5)
**Employment status**	Student	539 (33.4)
	Regular work	837 (51.8)
	Unemployed/ irregular work	239 (14.8)
**Health worker**	Yes	120 (7.4)
	No	1495 (92.6)
**Actual Lockdown**	Yes	1439 (89.1)
	No	176 (10.9)
**Recent quarantine in the past 14 days**	Yes	113 (7)
	No	1502 (93)
**Information received**	Sufficient	1035 (64.1)
	Insufficient	580 (35.9)
**Social media exposure**	Less	243 (15)
	Sometimes	772 (47.8)
	Frequently	600 (37.2)
**Times to focus on the COVID-19**	<1 hour	380 (23.5)
	1-2 hours	785 (48.6)
	≥3 hours	450 (27.9)
**Recent traumatic event in the past 14days**	Yes	794 (49.2)
	No	821 (50.8)
**Consultation with doctor in the clinic in the past 14 days**	Yes	137 (8.5)
	No	1478 (91.5)
**Recent physical symptom in the past 14 days**	No physical symptom	954 (59.1)
	At least one of the physical symptoms	661 (40.9)
	Fever	40 (2.5)
	Chills	99 (6.1)
	Headache	369 (22.8)
	Myalgia	164 (10.2)
	Cough	138 (8.5)
	Dyspnea	103 (6.4)
	Dizziness	164 (10.2)
	Other	13 (0.8)

SD; standard deviation**s** n; number

**Prevalence of anxiety, depressive symptoms and insomnia:** a considerable proportion of participants had symptoms of anxiety (1145 [70.9%]), depression (1149 [71.1%]), and insomnia (978 [60.6%]). The prevalence of anxiety case and depression case was 44.7% and 40% respectively. Clinical insomnia was reported in 31.3% of participants (moderate 23.9%; severe 7.4%).

### Factors associated with anxiety, depressive symptoms and insomnia

**Anxiety symptoms associated factors:** the results of univariable and multivariable analyses to determine the factors correlated to anxiety symptoms were summarized in Table 2. Many factors were associated with anxiety symptoms in univariable analysis: female gender, having organic diseases, history of mental illness, alcohol consumption, health workers (negative association), actual lockdown, insufficient information received among the pandemic, frequently social media exposure, times to focus on COVID-19 ≥ 3hours, consultation with doctor in the clinic in the past 14 days, recent physical symptom in the past 14 days and recent traumatic event in the past 14 days. Logistic regression analyses showed that, after controlling for confounders, anxiety symptoms were correlated to female gender 1.784, 95% CI: 1.252-2.542; p = 10^-3^, history of mental illness 1.680, 95% CI: 1.329-2.125; p <10^-3^ alcohol consumption 1.437, 95% CI: 1.077-1.916; p=0.014, health workers (negative association) 0.584, 95% CI: 0.438-0.780; p <10^-3^, insufficient information received among the pandemic 1.264, 95% CI: 1.016-1.574; p = 0.036, frequently social media exposure 1.578, 95% CI: 1.250-1.992; p <10^-3^, times to focus on COVID-19 ≥ 3hours 1.840, 95% CI: 1.433-2.362; p <10^-3^, consultation with doctor in the clinic in the past 14 days 1.821, 95% CI: 1.220-2.718; p= 0.003 , recent physical symptom in the past 14 days 1.700, 95% CI: 1.363-2.121; p <10^-3^and recent traumatic event in the past 14 days 1.641,95% CI: 1.331-2.024; p <10^-3^.

**Table 2:** anxiety symptoms associated factors

	Univariable analysis		Multivariable analysis	
	aOR (95% CI)	p Value	aOR (95% CI)	p Value
**Age <35 years**	1.072 (0.862 - 1.333)	0.53		
**Female**	1.624 (1.177 - 2.241)	0.003	1.784 (1.252 - 2.542)	10^-3^
**Unmarried**	1.074 (0.877 - 1.316)	0.49		
**High school level**	0.892 (0.589 - 1.352)	0.59		
**Having organic diseases**	1.449 (1.118 - 1.876)	0.005	0.871 (0.660 - 1.150)	0.33
**History of mental illness**	2.014 (1.617 - 2.510)	<10^-3^	1.680 (1.329 - 2.125)	<10^-3^
**Smoking**	1.212 (0.973 - 1.510)	0.86		
**Alcohol consumption**	1.350 (1.038 - 1.756)	0.025	1.437 (1.077 - 1.916)	0.014
**Students**	1.070 (0.869 - 1.317)	0.52		
**Health worker**	0.621 (0.473 - 0.815)	10^-3^	0.584 (0.438 - 0.780)	<10^-3^
**Actual Lockdown**	1.396 (1.012 - 1.927)	0.042	1.218 (0.794 - 1.870)	0.36
**Recent quarantine in the past 14 days**	0.781 (0.532 - 1.144)	0.26		
**Insufficient information received**	1.294 (1.055 - 1.587)	0.013	1.264 (1.016 - 1.574)	0.036
**Frequently Social media exposure**	1.669 (1.362 - 2.046)	<10^-3^	1.578 (1.250 - 1.992)	<10^-3^
**Times to focus on COVID-19 ≥ 3hours**	2.163 (1.734 - 2.699)	<10^-3^	1.840 (1.433 - 2.362)	<10^-3^
**Consultation with doctor in the clinic in the past 14 days**	2.564 (1.776 - 3.703)	<10^-3^	1.821 (1.220 - 2.718)	0.003
**Recent physical symptom in the past 14 days**	2.174 (1.776 - 2.659)	<10^-3^	1.700 (1.363 - 2.121)	<10^-3^
**Recent traumatic event in the past 14 days**	1.655 (1.358 - 2.016)	<10^-3^	1.641 (1.331 - 2.024)	<10^-3^

**aOR**; adjusted odds ratio **CI**; confidence interval

**Depressive symptoms associated factors:** univariable analysis revealed that depressive symptoms were associated with female gender, history of mental illness, smoking, students, actual lockdown, insufficient information received among the pandemic, frequently social media exposure, times to focus on COVID-19 ≥ 3hours, consultation with doctor in the clinic in the past 14 days, recent physical symptom in the past 14 days and recent traumatic event in the past 14 days. After controlling for confounders by logistic regression analyses, depressive symptoms were correlated to female gender 1.637, 95% CI: 1.150-2.331; p= 0.006, history of mental illness 1.498, 95% CI: 1.189-1.888; p= 10^-3^, smoking 1.317, 95% CI: 1.038-1.673; p=0.024, students 1.549, 95% CI: 1.242-1.932; p <10^-3^, times to focus on COVID-19 ≥ 3hours 1.956, 95% CI: 1.555-2.461; p <10^-3^, recent physical symptom in the past 14 day 1.576, 95% CI: 1.275-1.948; p <10^-3^and recent traumatic event in the past 14 days 1.558, 95% CI: 1.265-1.919; p <10^-3^. The results of univariable and multivariable analyses to identify depressive symptoms associated factors were summarized in Table 3.

**Table 3:** depressive symptoms associated factors

	Univariable analysis		Multivariable Analysis	
	aOR (95% CI)	p Value	aOR (95% CI)	p Value
Age <35 years	1.136 (0.910 - 1.418)	0.26		
Female	1.635 (1.172 - 2.282)	0.004	1.637 (1.150 - 2.331)	0.006
Unmarried	0.883 (0.718 - 1.086)	0.24		
High school level	0.727 (0.480 - 1.101)	0.31		
Having organic diseases	0.802 (0.618 - 1.040)	0.96		
History of mental illness	1.783 (1.432 - 2.220)	<10^-3^	1.498 (1.189 - 1.888)	10^-3^
Smoking	1.278 (1.024-1.595)	0.03	1.317 (1.038-1.673)	0.024
Alcohol consumption	1.208 (0.926-1.575)	0.63		
Students	1.452 (1.177-1.791)	<10^-3^	1.549 (1.242-1.932)	<10^-3^
Health worker	0.782 (0.595 – 1.020)	0.77		
Actual lockdown	1.447 (1.037 - 2.018)	0.029	1.023 (0.673 - 1.554)	0.916
Recent quarantine in the past 14 days	0.684 (0.466 - 1.004)	0.51		
Insufficient information received	1.336 (1.087 - 1.643)	0.006	1.225 (0.987 - 1.519)	0.066
Frequently social media exposure	1.246 (1.015 –1.530)	0.036	1.090 (0.864 - 1.376)	0.465
Times to focus on COVID-19 ≥ 3hours	1.931 (1.549 - 2.407)	<10^-3^	1.956 (1.555 - 2.461)	<10^-3^
Consultation with doctor in the clinic in the past 14 days	1.485 (1.046 - 2.109)	0.026	1.113 (0.761 - 1.629)	0.58
Recent physical symptom in the past 14 days	1.789 (1.459 - 2.193)	<10^-3^	1.576 (1.275 - 1.948)	<10^-3^
Recent traumatic event in the past 14 days	1.601 (1.311 - 1.956)	<10^-3^	1.558 (1.265 - 1.919)	<10^-3^

**aOR**; adjusted odds ratio**,CI**; confidence interval

**Insomnia associated factors:** several factors were associated with clinical insomnia in univariable analysis: younger (age <35 years), female gender, being unmarried, having organic diseases, history of mental illness, smoking, alcohol consumption, students, health workers (negative association), actual lockdown, recent quarantine in the past 14 days, insufficient information received among the pandemic, times to focus on COVID-19 ≥3hours, recent physical symptom in the past 14 days and recent traumatic event in the past 14 days. Logistic regression analyses showed that, after controlling for confounders, clinical insomnia was correlated to younger (age < 35 years) 1.592, 95% CI: 1.17 -2.152; p= 0.003, female gender 1864, 95% CI: 1.252-2.775; p= 0.002, having organic diseases 1.527, 95% CI: 1.131-2.061; p= 0.006, history of mental illness 1.777, 95% CI: 1.396-2.263; p <10^-3^, smoking 1.560, 95% CI: 1.163-2.094; p = 0.003, students 1.931, 95% CI: 1.495-2.495; p <10^-3^, health workers (negative association) 0.594, 95% CI: 0.430-0.819; p = 10^-3^, times to focus on COVID-19 ≥ 3hours 1.877, 95% CI: 1.467-2.400; p <10^-3^and recent traumatic event in the past 14 days 1.431, 95% CI: 1.144-1.789; p = 0.002. The results of all analyses to determine factors associated with insomnia were summarized in Table 4.

**Table 4:** insomnia associated factors

	Univariable analysis		Multivariable analysis	
	aOR (95% CI)	p Value	aOR (95% CI)	p Value
Age <35 years	1.791 (1.396 - 2.298)	<10^-3^	1.592 (1.177 - 2.152)	0.003
Female	1.781 (1.229 - 2.580)	0.002	1864 (1.252 - 2.775)	0.002
Unmarried	1.808 (1.438 - 2.267)	<10^-3^	1.220 (0.900 - 1.652)	0.2
High school level	0.806 (0.522 - 1.242)	0.32		
Having organic diseases	1.360 (1.038 - 1.779)	0.025	1.527 (1.131 - 2.061)	0.006
History of mental illness	2.141 (1.707 - 2.685)	<10^-3^	1.777 (1.396 - 2.263)	<10^-3^
Smoking	1.579 (1.254 - 1.987)	<10^-3^	1.560 (1.163 - 2.094)	0.003
Alcohol consumption	1.690 (1.290 - 2.215)	<10^-3^	1.351 (0.958 - 1.905)	0.086
Students	2.115 (1.699 - 2.632)	<10^-3^	1.931 (1.495 - 2.495)	<10^-3^
Health worker	0.592 (0.435 - 0.805)	10^-3^	0.594 (0.430 - 0.819)	10^-3^
Actual Lockdown	2.398 (1.595 - 3.606)	<10^-3^	0.682 (0.423 - 1.100)	0.116
Recent quarantine in the past 14 days	1.824 (1.240 - 2.688)	0.002	0.820 (0.532 - 1,264)	0.368
Insufficient information received	1.339 (1.078 - 1.664)	0.008	1.180 (0.937 - 1.487)	0.159
Frequently Social media exposure	1.055 (0.849 - 1.311)	0.62		
Times to focus on COVID-19 ≥ 3hours	1.567 (1.247 - 1.969)	<10^-3^	1.877 (1.467 - 2.400)	<10^-3^
Consultation with doctor in the clinic in the past 14 days	0.774 (0.537 - 1.115)	0.61		
Recent physical symptom in the past 14 days	1.414 (1.143 - 1.748)	10^-3^	1.189 (0.945 - 1.496)	0.139
Recent traumatic event in the past 14 days	1.477 (1.194 - 1.824)	<10^-3^	1.431 (1.144 - 1.789)	0.002

## Discussion

This is the first study assessing the psychological impact on Tunisian citizens during the peak of the COVID-19 pandemic when strict lockdown measures were in place. This cross-sectional survey revealed high prevalence of mental health symptoms during this period. Many factors were significantly associated with anxiety symptoms, depressive symptoms and insomnia: female, students, smoking or drinking habit, history of mental illness, spending ≥3 hours per day focusing on the COVID-19 epidemic, having physical symptoms or experiencing a recent traumatic event. Moreover, having insufficient information among COVID-19 pandemic was correlated to experiencing anxiety symptoms. Nearly half of participants presented anxiety/depression case (44.7% and 40% respectively) and almost one third of them (31.3%) experienced clinical insomnia. There are few studies on mental health among the general population in Tunisia. One study on Tunisian population published in 2017 underlined a prevalence of 33% and 31% of anxiety and depressive symptoms respectively [15]. A recent meta-analysis of the impact of the COVID-19 pandemic on mental health in Asian countries revealed that anxiety and depression are often more than 20% prevalent [16], which is similar to the results of a recent Spanish study [17]. Compared to other recently published studies, our results are higher, although close to the Italian results (57.1% poor sleep quality, 32.1% generalized anxiety symptoms, 41.8% psychological distress) [18]. However, statistical comparison is impossible considering the different characteristics of the samples and the variety of questionnaires used in the studies. All the same, our results clearly show the severity of negative psychological impact on Tunisian citizens during the COVID-19 pandemic with strict lockdown measures.

Our socio-demographic data indicate that female have experienced a greater psychological impact of the COVID-19 pandemic as well as higher levels of anxiety, depressive symptoms and insomnia. This finding was also objectified in multiple recent studies [16,18-20] and it can support the already established gender gap for anxious and depressive symptoms [21,22]. In addition, younger people (<35years) had an increased risk of developing sleep disturbances. Our results were similar to those of two previous studies in China and in Italy during COVID-19 pandemic [18,23]. Huang *et al*. have also shown that young people were more likely to develop anxiety and depressive symptoms [24]. Students were more likely to develop depressive symptoms and insomnia. These findings are in agreement with other studies [17,25,26]. During the period of this study, Tunisian universities closed their doors and students have stopped their studies. The development by educational authorities of web applications to provide courses or other teaching activities could help these students to complete their training [27]. In our study, smoking participants had an increased risk of developing depressive symptoms and insomnia while responders who consumed alcohol were more likely to experience anxiety symptoms. Smoking incidence and maintenance generalize across various emotional disorders, including depression [15] and anxiety disorders [28]. In the same way, the co-occurrence of anxiety disorders and alcohol use is relatively common. Cumulative evidence from epidemiological and clinical studies over the past few decades has highlighted both the frequency and clinical impact of this comorbidity [29].

Participants with history of mental illness were significantly more likely to experience anxiety, depressive symptoms and insomnia which was similarly objectified in a recent Chinese study [30]. This fragile population may be at risk of relapse following the pandemic [31]. Containment measures and cessation of non-urgent medical consultations made it difficult for these patients to see their referrers and to have renewal of medical prescriptions [32]. Faced with this situation, doctors must be more vigilant in psychiatric emergencies so that appropriate and timely interventions can be carried out. In addition, the continued availability of health services and essential drugs is essential. Telemedicine consultation should also be practiced and encouraged. Healthcare workers had lower risk of anxiety and insomnia. Although this finding is different from previous studies [16,33,34], it seems to be in agreement with the results of Jungmann *et al*.’s study [20]. This finding could be explained by the fact that health workers are better informed and are confronted with the problem of COVID-19 in their daily work, which could potentially lead to habituation effect and lower degree of anxiety [20]. Targeted studies involving a larger number of health care workers are necessary in order to have conclusive results. In line with recent studies [17,24,26,35], we found that frequently social media exposure and feeling insufficient the information received among COVID-19 pandemic were associated with developing anxiety symptoms. In addition, we found that people who spent too much time (≥ 3 hours) focusing on the COVID-19 were more likely to experience anxiety, depressive symptoms and insomnia. During COVID-19 pandemic, there is an important increase in connectivity between people and societies. Several false reports were disseminated which raise unfounded fears [26,36]. People should be aware of the importance of accessing official information sources to avoid fake news and should request health professionals for advice if necessary.

Our results revealed that people who recently presented physical symptoms or consulted a doctor, experienced higher levels of anxiety and depressive symptoms. These findings were similar to those of recent studies [17,19]. This emphasizes the necessity of early intervention for people with physical symptoms. As their presentation to hospital, health professionals should take the opportunity to provide them advice and resources for psychological support and interventions. Our study showed that having experienced a traumatic event in the past 14 days was correlated to anxiety, depressive symptoms and insomnia. Previous studies reported a negative impact on the mental health of those who underwent traumatic events, such as those related to the global spread of unknown epidemics [37,38], which could culminate in post-traumatic symptomatology [39]. Although this study presents the first data describing the psychological impact of the COVID-19 pandemic in Tunisian general population, some limitations must be pointed out. The using of an online survey limits the generalizability of the results, since some subjects did not have internet access. However, in these particular epidemiological conditions requiring social distancing measures, this method represents the best solution for data collection. Moreover, the absence of data indicating the prevalence of considered dimensions, in particular anxiety, depression and sleep disorders in representative samples of the general Tunisian population before the COVID-19 pandemic, did not allow us to compare our results or to highlight an increase in the prevalence of these diseases during this pandemic.

## Conclusion

The results of this study provide the first data about the psychological impact of the COVID-19 pandemic carried out in the whole Tunisian territory. Our study revealed a high prevalence of anxiety/depressive symptoms and insomnia during this critic period which was associated with several factors as female gender, students, smoking or drinking habit, history of mental illness, having insufficient information among COVID-19 pandemic, spending ≥ 3 hours /day focusing on this pandemic, having physical symptoms or experiencing a recent traumatic event. Some appropriate interventions can be recommended to prevent mental health problems during the probable next waves of the COVID-19 pandemic. First, various media outlets should ensure the validity of the information before disseminating it in order to alleviate the impact of fake news on mental health among people. Besides, health authorities should establish an official platform integrating all the necessary information in relation to the pandemic with offer of mental health counseling. It is also possible to provide people online interventions aimed at raising their awareness of the psychological impact of pandemics. Adaptive emotional regulation strategies in the form of evaluated online tools may be a promising way to reduce these psychological problems, especially in vulnerable populations.

### What is known about this topic

- Apart from its physical burden on patients, COVID-19 has enormous psychological impact;
- Prior research showed a wide range of psychosocial responses in this kind of global pandemic, like anxiety and depressive symptoms as well as sleep disturbances.

### What this study adds

- Tunisians presented a high prevalence of anxiety, depressive symptoms and insomnia, especially vulnerable population such as women, students, people with history of mental illness and those having physical symptoms or having experienced a recent traumatic event;
- Frequently social media exposure and feeling having insufficient information were predictors of anxiety symptoms;
- The study of risk factors and moderators can help to better understand the development and maintenance of psychological symptoms and to develop preventive measures and targeted therapeutic interventions
